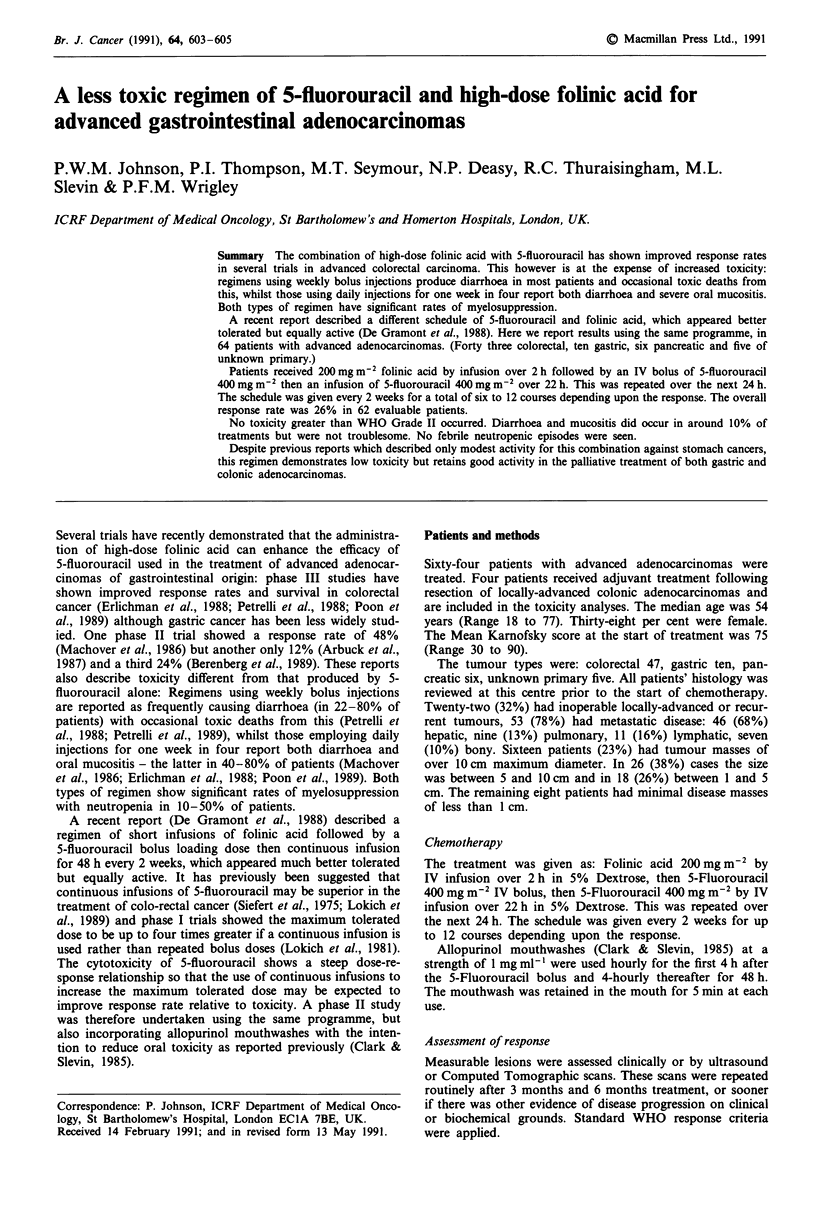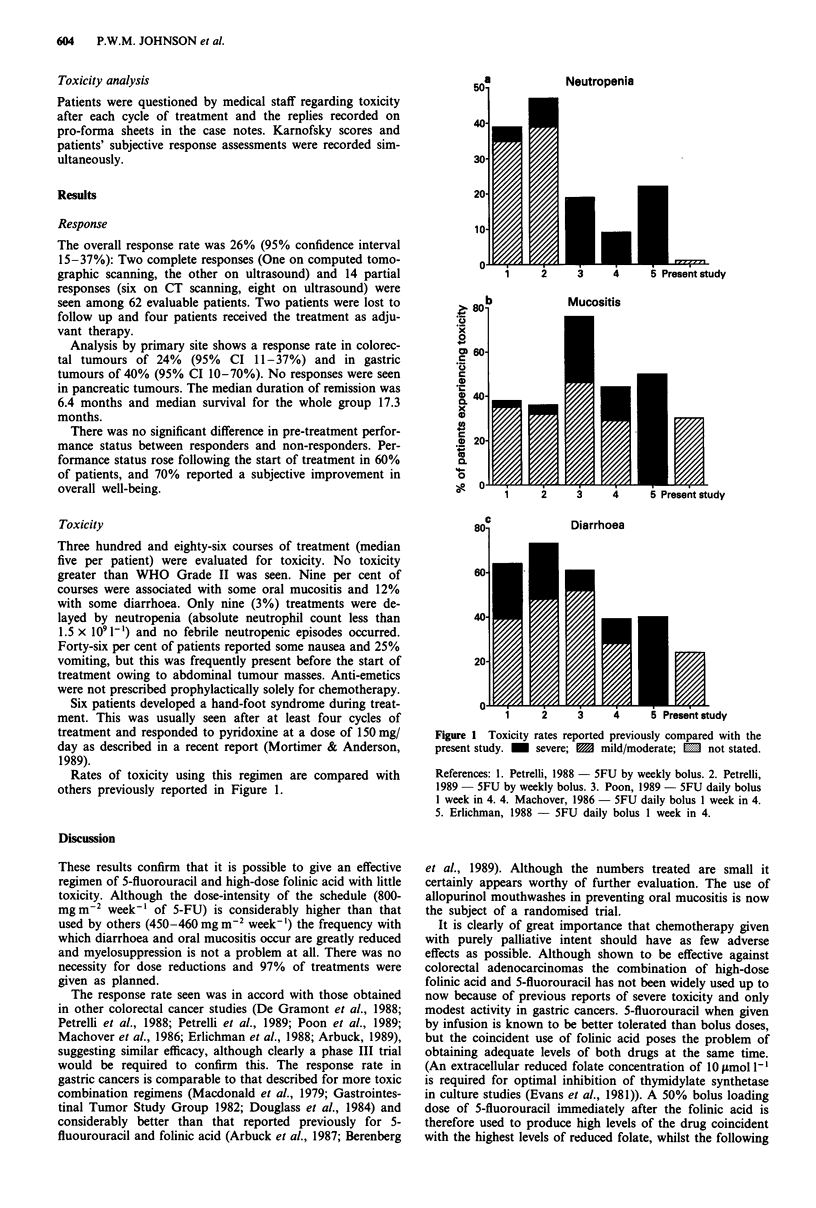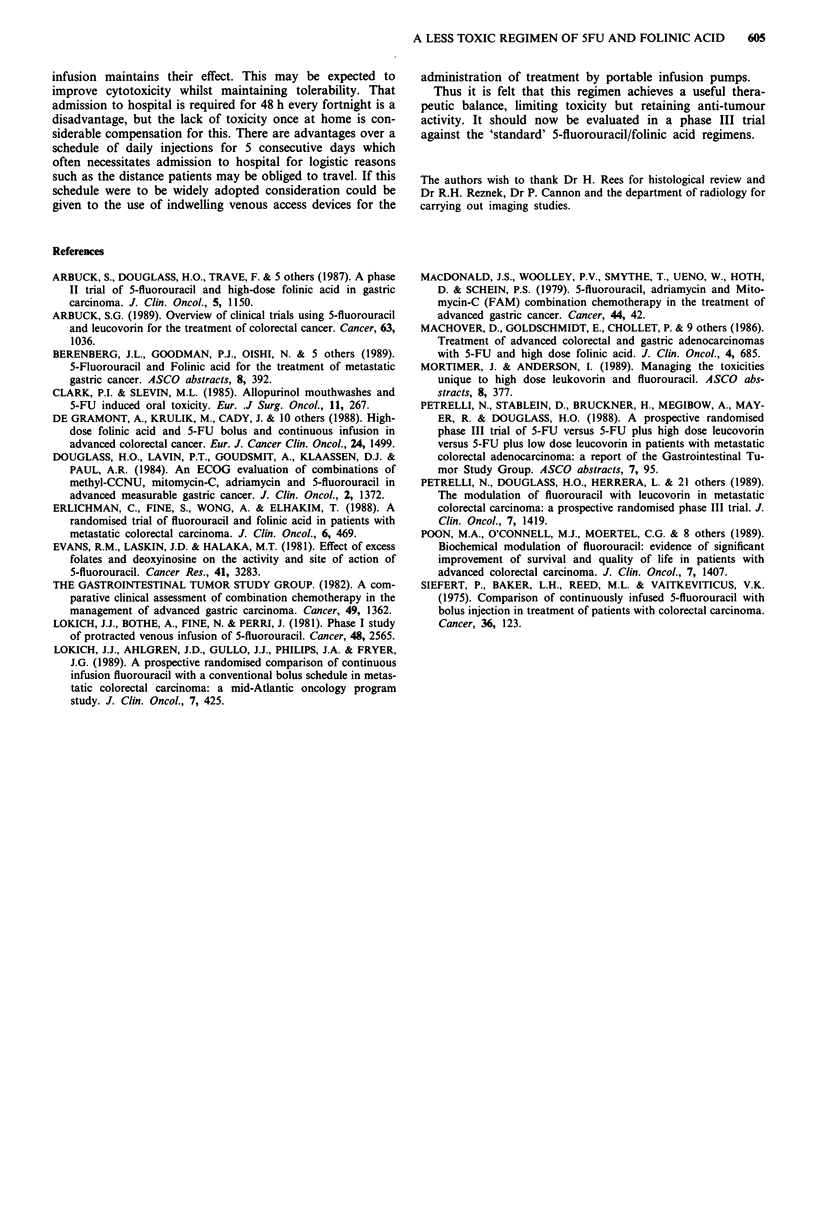# A less toxic regimen of 5-fluorouracil and high-dose folinic acid for advanced gastrointestinal adenocarcinomas.

**DOI:** 10.1038/bjc.1991.358

**Published:** 1991-09

**Authors:** P. W. Johnson, P. I. Thompson, M. T. Seymour, N. P. Deasy, R. C. Thuraisingham, M. L. Slevin, P. F. Wrigley

**Affiliations:** ICRF Department of Medical Oncology, St Bartholomew's Hospital, London, UK.

## Abstract

The combination of high-dose folinic acid with 5-fluorouracil has shown improved response rates in several trials in advanced colorectal carcinoma. This however is at the expense of increased toxicity: regimens using weekly bolus injections produce diarrhoea in most patients and occasional toxic deaths from this, whilst those using daily injections for one week in four report both diarrhoea and severe oral mucositis. Both types of regimen have significant rates of myelosuppression. A recent report described a different schedule of 5-fluorouracil and folinic acid, which appeared better tolerated but equally active (De Gramont et al., 1988). Here we report results using the same programme, in 64 patients with advanced adenocarcinomas. (Forty three colorectal, ten gastric, six pancreatic and five of unknown primary.) Patients received 200 mg m-2 folinic acid by infusion over 2 h followed by an IV bolus of 5-fluorouracil 400 mg m-2 then an infusion of 5-fluorouracil 400 mg m-2 over 22 h. This was repeated over the next 24 h. The schedule was given every 2 weeks for a total of six to 12 courses depending upon the response. The overall response rate was 26% in 62 evaluable patients. No toxicity greater than WHO Grade II occurred. Diarrhoea and mucositis did occur in around 10% of treatments but were not troublesome. No febrile neutropenic episodes were seen. Despite previous reports which described only modest activity for this combination against stomach cancers, this regimen demonstrates low toxicity but retains good activity in the palliative treatment of both gastric and colonic adenocarcinomas.


Br. J. Cancer (1991), 64, 603-605 © Macmillan Press Ltd., 1991

### A less toxic regimen of 5-fluorouracil and high-dose folinic acid for advanced gastrointestinal adenocarcinomas

P.W.M. Johnson, P.I. Thompson, M.T. Seymour, N.P. Deasy, R.C. Thuraisingham, M.L. Slevin & P.F.M. Wrigley

*ICRF Department of Medical Oncology, St Bartholomew's and Homerton Hospitals, London, UK.*

**Summary** The combination of high-dose folinic acid with 5-fluorouracil has shown improved response rates in several trials in advanced colorectal carcinoma. This however is at the expense of increased toxicity: regimens using weekly bolus injections produce diarrhoea in most patients and occasional toxic deaths from this, whilst those using daily injections for one week in four report both diarrhoea and severe oral mucositis. Both types of regimen have significant rates of myelosuppression.

A recent report described a different schedule of 5-fluorouracil and folinic acid, which appeared better tolerated but equally active (De Gramont *et al*., 1988). Here we report results using the same programme, in 64 patients with advanced adenocarcinomas. (Forty three colorectal, ten gastric, six pancreatic and five of unknown primary.)

Patients received 200 mg m$^{-2}$ folinic acid by infusion over 2 h followed by an IV bolus of 5-fluorouracil 400 mg m$^{-2}$ then an infusion of 5-fluorouracil 400 mg m$^{-2}$ over 22 h. This was repeated over the next 24 h. The schedule was given every 2 weeks for a total of six to 12 courses depending upon the response. The overall response rate was 26% in 62 evaluable patients.

No toxicity greater than WHO Grade II occurred. Diarrhoea and mucositis did occur in around 10% of treatments but were not troublesome. No febrile neutropenic episodes were seen.

Despite previous reports which described only modest activity for this combination against stomach cancers, this regimen demonstrates low toxicity but retains good activity in the palliative treatment of both gastric and colonic adenocarcinomas.

Several trials have recently demonstrated that the administration of high-dose folinic acid can enhance the efficacy of 5-fluorouracil used in the treatment of advanced adenocarcinomas of gastrointestinal origin: phase III studies have shown improved response rates and survival in colorectal cancer (Erlichman *et al*., 1988; Petrelli *et al*., 1988; Poon *et al*., 1989) although gastric cancer has been less widely studied. One phase II trial showed a response rate of 48% (Machover *et al*., 1986) but another only 12% (Arbuck *et al*., 1987) and a third 24% (Berenberg *et al*., 1989). These reports also describe toxicity different from that produced by 5-fluorouracil alone: Regimens using weekly bolus injections are reported as frequently causing diarrhoea (in 22-80% of patients) with occasional toxic deaths from this (Petrelli *et al*., 1988; Petrelli *et al*., 1989), whilst those employing daily injections for one week in four report both diarrhoea and oral mucositis - the latter in 40-80% of patients (Machover *et al*., 1986; Erlichman *et al*., 1988; Poon *et al*., 1989). Both types of regimen show significant rates of myelosuppression with neutropenia in 10-50% of patients.

A recent report (De Gramont *et al*., 1988) described a regimen of short infusions of folinic acid followed by a 5-fluorouracil bolus loading dose then continuous infusion for 48 h every 2 weeks, which appeared much better tolerated but equally active. It has previously been suggested that continuous infusions of 5-fluorouracil may be superior in the treatment of colo-rectal cancer (Siefert *et al*., 1975; Lokich *et al*., 1989) and phase I trials showed the maximum tolerated dose to be up to four times greater if a continuous infusion is used rather than repeated bolus doses (Lokich *et al*., 1981). The cytotoxicity of 5-fluorouracil shows a steep dose-response relationship so that the use of continuous infusions to increase the maximum tolerated dose may be expected to improve response rate relative to toxicity. A phase II study was therefore undertaken using the same programme, but also incorporating allopurinol mouthwashes with the intention to reduce oral toxicity as reported previously (Clark & Slevin, 1985).

Correspondence: P. Johnson, ICRF Department of Medical Oncology, St Bartholomew's Hospital, London EC1A 7BE, UK.
Received 14 February 1991; and in revised form 13 May 1991.

### Patients and methods

Sixty-four patients with advanced adenocarcinomas were treated. Four patients received adjuvant treatment following resection of locally-advanced colonic adenocarcinomas and are included in the toxicity analyses. The median age was 54 years (Range 18 to 77). Thirty-eight per cent were female. The Mean Karnofsky score at the start of treatment was 75 (Range 30 to 90).

The tumour types were: colorectal 47, gastric ten, pancreatic six, unknown primary five. All patients' histology was reviewed at this centre prior to the start of chemotherapy. Twenty-two (32%) had inoperable locally-advanced or recurrent tumours, 53 (78%) had metastatic disease: 46 (68%) hepatic, nine (13%) pulmonary, 11 (16%) lymphatic, seven (10%) bony. Sixteen patients (23%) had tumour masses of over 10 cm maximum diameter. In 26 (38%) cases the size was between 5 and 10 cm and in 18 (26%) between 1 and 5 cm. The remaining eight patients had minimal disease masses of less than 1 cm.

### *Chemotherapy*

The treatment was given as: Folinic acid 200 mg m$^{-2}$ by IV infusion over 2 h in 5% Dextrose, then 5-Fluorouracil 400 mg m$^{-2}$ IV bolus, then 5-Fluorouracil 400 mg m$^{-2}$ by IV infusion over 22 h in 5% Dextrose. This was repeated over the next 24 h. The schedule was given every 2 weeks for up to 12 courses depending upon the response.

Allopurinol mouthwashes (Clark & Slevin, 1985) at a strength of 1 mg ml$^{-1}$ were used hourly for the first 4 h after the 5-Fluorouracil bolus and 4-hourly thereafter for 48 h. The mouthwash was retained in the mouth for 5 min at each use.

### *Assessment of response*

Measurable lesions were assessed clinically or by ultrasound or Computed Tomographic scans. These scans were repeated routinely after 3 months and 6 months treatment, or sooner if there was other evidence of disease progression on clinical or biochemical grounds. Standard WHO response criteria were applied.

### *Toxicity analysis*

Patients were questioned by medical staff regarding toxicity after each cycle of treatment and the replies recorded on pro-forma sheets in the case notes. Karnofsky scores and patients' subjective response assessments were recorded simultaneously.

### Results

### *Response*

The overall response rate was 26% (95% confidence interval 15–37%): Two complete responses (One on computed tomographic scanning, the other on ultrasound) and 14 partial responses (six on CT scanning, eight on ultrasound) were seen among 62 evaluable patients. Two patients were lost to follow up and four patients received the treatment as adjuvant therapy.

Analysis by primary site shows a response rate in colorectal tumours of 24% (95% CI 11–37%) and in gastric tumours of 40% (95% CI 10–70%). No responses were seen in pancreatic tumours. The median duration of remission was 6.4 months and median survival for the whole group 17.3 months.

There was no significant difference in pre-treatment performance status between responders and non-responders. Performance status rose following the start of treatment in 60% of patients, and 70% reported a subjective improvement in overall well-being.

### *Toxicity*

Three hundred and eighty-six courses of treatment (median five per patient) were evaluated for toxicity. No toxicity greater than WHO Grade II was seen. Nine per cent of courses were associated with some oral mucositis and 12% with some diarrhoea. Only nine (3%) treatments were delayed by neutropenia (absolute neutrophil count less than $1.5 \times 10^9 l^{-1}$) and no febrile neutropenic episodes occurred. Forty-six per cent of patients reported some nausea and 25% vomiting, but this was frequently present before the start of treatment owing to abdominal tumour masses. Anti-emetics were not prescribed prophylactically solely for chemotherapy.

Six patients developed a hand-foot syndrome during treatment. This was usually seen after at least four cycles of treatment and responded to pyridoxine at a dose of 150 mg/day as described in a recent report (Mortimer & Anderson, 1989).

Rates of toxicity using this regimen are compared with others previously reported in Figure 1.

**Figure 1** Toxicity rates reported previously compared with the present study. ■ severe; ▨ mild/moderate; ▩ not stated.

References: 1. Petrelli, 1988 — 5FU by weekly bolus. 2. Petrelli, 1989 — 5FU by weekly bolus. 3. Poon, 1989 — 5FU daily bolus 1 week in 4. 4. Machover, 1986 — 5FU daily bolus 1 week in 4. 5. Erlichman, 1988 — 5FU daily bolus 1 week in 4.

### Discussion

These results confirm that it is possible to give an effective regimen of 5-fluorouracil and high-dose folinic acid with little toxicity. Although the dose-intensity of the schedule (800-mg $m^{-2}$ week$^{-1}$ of 5-FU) is considerably higher than that used by others (450–460 mg $m^{-2}$ week$^{-1}$) the frequency with which diarrhoea and oral mucositis occur are greatly reduced and myelosuppression is not a problem at all. There was no necessity for dose reductions and 97% of treatments were given as planned.

The response rate seen was in accord with those obtained in other colorectal cancer studies (De Gramont *et al.*, 1988; Petrelli *et al.*, 1988; Petrelli *et al.*, 1989; Poon *et al.*, 1989; Machover *et al.*, 1986; Erlichman *et al.*, 1988; Arbuck, 1989), suggesting similar efficacy, although clearly a phase III trial would be required to confirm this. The response rate in gastric cancers is comparable to that described for more toxic combination regimens (Macdonald *et al.*, 1979; Gastrointestinal Tumor Study Group 1982; Douglass *et al.*, 1984) and considerably better than that reported previously for 5-fluourouracil and folinic acid (Arbuck *et al.*, 1987; Berenberg *et al.*, 1989). Although the numbers treated are small it certainly appears worthy of further evaluation. The use of allopurinol mouthwashes in preventing oral mucositis is now the subject of a randomised trial.

It is clearly of great importance that chemotherapy given with purely palliative intent should have as few adverse effects as possible. Although shown to be effective against colorectal adenocarcinomas the combination of high-dose folinic acid and 5-fluorouracil has not been widely used up to now because of previous reports of severe toxicity and only modest activity in gastric cancers. 5-fluorouracil when given by infusion is known to be better tolerated than bolus doses, but the coincident use of folinic acid poses the problem of obtaining adequate levels of both drugs at the same time. (An extracellular reduced folate concentration of 10 $\mu$mol $l^{-1}$ is required for optimal inhibition of thymidylate synthetase in culture studies (Evans *et al.*, 1981)). A 50% bolus loading dose of 5-fluorouracil immediately after the folinic acid is therefore used to produce high levels of the drug coincident with the highest levels of reduced folate, whilst the following infusion maintains their effect. This may be expected to improve cytotoxicity whilst maintaining tolerability. That admission to hospital is required for 48 h every fortnight is a disadvantage, but the lack of toxicity once at home is considerable compensation for this. There are advantages over a schedule of daily injections for 5 consecutive days which often necessitates admission to hospital for logistic reasons such as the distance patients may be obliged to travel. If this schedule were to be widely adopted consideration could be given to the use of indwelling venous access devices for the administration of treatment by portable infusion pumps.

Thus it is felt that this regimen achieves a useful therapeutic balance, limiting toxicity but retaining anti-tumour activity. It should now be evaluated in a phase III trial against the 'standard' 5-fluorouracil/folinic acid regimens.

The authors wish to thank Dr H. Rees for histological review and Dr R.H. Reznek, Dr P. Cannon and the department of radiology for carrying out imaging studies.

**References**

ARBUCK, S., DOUGLASS, H.O., TRAVE, F. & 5 others (1987). A phase II trial of 5-fluorouracil and high-dose folinic acid in gastric carcinoma. *J. Clin. Oncol.*, **5**, 1150.

ARBUCK, S.G. (1989). Overview of clinical trials using 5-fluorouracil and leucovorin for the treatment of colorectal cancer. *Cancer*, **63**, 1036.

BERENBERG, J.L., GOODMAN, P.J., OISHI, N. & 5 others (1989). 5-Fluorouracil and Folinic acid for the treatment of metastatic gastric cancer. *ASCO abstracts*, **8**, 392.

CLARK, P.I. & SLEVIN, M.L. (1985). Allopurinol mouthwashes and 5-FU induced oral toxicity. *Eur. .J Surg. Oncol.*, **11**, 267.

DE GRAMONT, A., KRULIK, M., CADY, J. & 10 others (1988). High-dose folinic acid and 5-FU bolus and continuous infusion in advanced colorectal cancer. *Eur. J. Cancer Clin. Oncol.*, **24**, 1499.

DOUGLASS, H.O., LAVIN, P.T., GOUDSMIT, A., KLAASSEN, D.J. & PAUL, A.R. (1984). An ECOG evaluation of combinations of methyl-CCNU, mitomycin-C, adriamycin and 5-fluorouracil in advanced measurable gastric cancer. *J. Clin. Oncol.*, **2**, 1372.

ERLICHMAN, C., FINE, S., WONG, A. & ELHAKIM, T. (1988). A randomised trial of fluorouracil and folinic acid in patients with metastatic colorectal carcinoma. *J. Clin. Oncol.*, **6**, 469.

EVANS, R.M., LASKIN, J.D. & HALAKA, M.T. (1981). Effect of excess folates and deoxyinosine on the activity and site of action of 5-fluorouracil. *Cancer Res.*, **41**, 3283.

THE GASTROINTESTINAL TUMOR STUDY GROUP. (1982). A comparative clinical assessment of combination chemotherapy in the management of advanced gastric carcinoma. *Cancer*, **49**, 1362.

LOKICH, J.J., BOTHE, A., FINE, N. & PERRI, J. (1981). Phase I study of protracted venous infusion of 5-fluorouracil. *Cancer*, **48**, 2565.

LOKICH, J.J., AHLGREN, J.D., GULLO, J.J., PHILIPS, J.A. & FRYER, J.G. (1989). A prospective randomised comparison of continuous infusion fluorouracil with a conventional bolus schedule in metastatic colorectal carcinoma: a mid-Atlantic oncology program study. *J. Clin. Oncol.*, **7**, 425.

MACDONALD, J.S., WOOLLEY, P.V., SMYTHE, T., UENO, W., HOTH, D. & SCHEIN, P.S. (1979). 5-fluorouracil, adriamycin and Mitomycin-C (FAM) combination chemotherapy in the treatment of advanced gastric cancer. *Cancer*, **44**, 42.

MACHOVER, D., GOLDSCHMIDT, E., CHOLLET, P. & 9 others (1986). Treatment of advanced colorectal and gastric adenocarcinomas with 5-FU and high dose folinic acid. *J. Clin. Oncol.*, **4**, 685.

MORTIMER, J. & ANDERSON, I. (1989). Managing the toxicities unique to high dose leukovorin and fluorouracil. *ASCO abstracts*, **8**, 377.

PETRELLI, N., STABLEIN, D., BRUCKNER, H., MEGIBOW, A., MAYER, R. & DOUGLASS, H.O. (1988). A prospective randomised phase III trial of 5-FU versus 5-FU plus high dose leucovorin versus 5-FU plus low dose leucovorin in patients with metastatic colorectal adenocarcinoma: a report of the Gastrointestinal Tumor Study Group. *ASCO abstracts*, **7**, 95.

PETRELLI, N., DOUGLASS, H.O., HERRERA, L. & 21 others (1989). The modulation of fluorouracil with leucovorin in metastatic colorectal carcinoma: a prospective randomised phase III trial. *J. Clin. Oncol.*, **7**, 1419.

POON, M.A., O'CONNELL, M.J., MOERTEL, C.G. & 8 others (1989). Biochemical modulation of fluorouracil: evidence of significant improvement of survival and quality of life in patients with advanced colorectal carcinoma. *J. Clin. Oncol.*, **7**, 1407.

SIEFERT, P., BAKER, L.H., REED, M.L. & VAITKEVITICUS, V.K. (1975). Comparison of continuously infused 5-fluorouracil with bolus injection in treatment of patients with colorectal carcinoma. *Cancer*, **36**, 123.